# Hashimoto’s thyroiditis worsens ovaries in polycystic ovary syndrome patients compared to Anti-Müllerian hormone levels

**DOI:** 10.1186/s12902-021-00706-9

**Published:** 2021-03-09

**Authors:** Aliye Nigar Serin, Özer Birge, Aysel Uysal, Süheyla Görar, Feyza Tekeli

**Affiliations:** 1 Department of Gynecology and Obstetrics, Karamanoğlu Mehmet Bey University Faculty of Medicine, Karaman, Turkey; 2 Department of Gynecology and Obstetrics, Akdeniz University Faculty of Medicine, Antalya, Turkey; 3 Department of Gynecology and Obstetrics, Antalya Training and Research Hospital, Antalya, Turkey; 4 Department of Endocrinology and Metabolism, Antalya Training and Research Hospital, Antalya, Turkey; 5 Department of Biochemistry, Antalya Training and Research Hospital, Antalya, Turkey

**Keywords:** Polycystic ovary syndrome, Anti-Müllerian hormone, Autoimmunity, Hashimoto’s thyroiditis

## Abstract

**Background:**

The human ovary is the target of autoimmune attack in cases of autoimmune disorders, which can cause ovarian dysfunction. Due to the higher prevalence of Hashimoto’s Thyroiditis (HT) in Polycystic Ovary Syndrome (PCOS) patients, we aimed to evaluate ovarian reserve and the effect of autoimmune exposure time on ovarian reserve in PCOS patients with HT by Anti-Müllerian hormone (AMH) levels.

**Methods:**

Forty-six PCOS patients and 46 PCOS with HT diagnosed patients who are between 18 and 35 years old were recruited for this study. Detailed medical histories were obtained from all participants. Polycystic ovary image was evaluated and antral follicles were counted by transvaginal ultrasound. Modified Ferriman Gallwey score, body mass index, waist/hip ratio of the patients were examined. Hormonal, biochemical profiles and AMH levels of the patients were evaluated during the early follicular phase. The data of both groups were statistically analyzed with SPSS 18.0.

**Results:**

20 (43.5%) patients in the PCOS group were fertile, 8 (17.4%) patients in the PCOS + HT group were fertile, fertility rate was significantly lower in PCOS + HT group. The mean AMH value was 8.8 ± 8.8 in the PCOS + HT group and 12.4 ± 8.1 in the PCOS group and it was significantly lower in the PCOS + HT group (*p* = 0.043). AMH values were significantly negatively correlated with anti-thyroid peroxidase antibody (anti-TPO) level and the duration of HT. There was a significant positive correlation between the anti-TPO level and the duration of HT.

**Conclusıon:**

We pointed out that the coexistence of PCOS and HT, two prevalent diseases of reproductive age, further diminished ovarian reserve. More exposure of the ovaries to autoantibodies can cause ovarian destruction, similar to the thyroid gland like HT. Because of all these close relations with PCOS and thyroid dysfunctions, we recommend evaluating both thyroid autoantibodies and hormone levels in PCOS patients at the first visit. Patients with PCOS + HT should be monitored more closely to determine the fertility treatment options and control premature ovarian failure (POF) table.

## Background

Polycystic ovary syndrome (PCOS) is one of the most common endocrine disorder affecting 6–8% of women of reproductive age and characterized by hyperandrogenism (HA) / hirsutism, oligo/amenorrhea, and polycystic ovaries (PCO) [1]. Although a variety of theories both genetic and environmental has been enounced for the etiopathogenesis, HA theory and the insulin resistance theory are highly accepted [2]. Inflammatory and autoimmune causes are also reported because of its close association with insulin resistance and thyroid disorders [3]. PCOS affects many body functions and is complicated with infertility, menstrual dysfunction, obesity, HA symptoms (such as hirsutism, acne, alopecia) and metabolic syndrome [4].

The prevalence of thyroid autoimmunity are reported higher in PCOS population than women in general [5–7]. Anti-thyroid peroxidase antibodies (anti-TPO) and anti thyroglobulin antibodies (anti-TG) are basic markers of thyroid autoimmunity as Hashimoto’s thyroiditis (HT) [8]. Variable studies demonstrated a robust correlation between thyroid antibodies and specific causes of infertility as PCOS, endometriosis and diminished ovarian reserve [9–11]. Poppe et al. reported that thyroid autoantibodies are significantly higher in infertile patients [12]. As it is a reason for medically treatable infertility, the close relationship between PCOS and autoimmune thyroid diseases seems to be crucial [13].

The state of the ovarian reserve is measured by anti-mullerian hormone (AMH) released from granulosa cells of antral follicles and decreasing with age [14]. Serum AMH levels indicate the number of follicles in the follicle pool and have been identified as a reliable marker for ovarian reserve [15, 16].

We aimed to investigate ovarian reserve and the effect of autoimmune exposure time on ovarian reserve in PCOS patients with HT by AMH levels.

## Methods

A total of 92 patients, aged 18–35 years, married, 46 PCOS patients and 46 patients who have both PCOS and HT, who applied to Antalya Training and Research Hospital’s obstetrics and gynecology outpatient clinic and endocrinology and metabolic diseases outpatient clinic were included in the study. Written and verbal informed consents were obtained from patients for the study, prepared in accordance with the Helsinki Declaration and accepted by the Antalya Training and Research Hospital Ethics Committee (Ethics Committee Approval date – No: 05.05.2016–9/11). This work was supported by the Scientific Research Projects Coordination Unit of Antalya Training and Research Hospital. The study was conducted single-centered.

### Patient selection and clinical measurements

The diagnosis of PCOS was made in the presence of at least any two of the Rotterdam criteria (oligo/amenorrhea, clinical and/or biochemical hyperandrogenism, and polycystic ovaries) after other possible etiological causes (congenital adrenal hyperplasia, androgen secreting tumor, Cushing syndrome) were excluded [17]. Oligo-anovulation was clinically determined by the presence of oligo-amenorrhea (fewer than 8 per year or absence of no bleeding for 3 months or more, excluding pregnancy). Hyperandrogenemia was diagnosed by evaluating testosterone (T), dehydroepiandrosterone sulfate (DHEA-S), androstenedione (A4) (the upper normal limits were total T = 0.89 ng/ml, A4 = 2.9 ng/ml, DHEAS =179 μg/dL). The presence of hirsutism, acne or alopecia was taken as the clinical determinant of hyperandrogenism. Hirsutism scores of the patients were determined using the modified Ferriman-Gallwey (mFG) scoring system. With this method, the hair density was scored between 0 and 4 in a total of nine anatomical regions: upper lip, chin, chest area, back, waist, lower and upper abdomen, upper parts of the arms and legs. Those with a total score of 8 or more were considered hirsute. In the ultrasonography, the PCO image was evaluated with the presence of more than 12 follicles in 2–9 mm dimensions with peripheral location or ovarian size > 10 cm^3^ volume. Ultrasonographic evaluation of the ovaries and antral follicle count (AFC) were performed transvaginally in the lithotomy position with MINDRAY brand DC-7 T model ultrasonography device. Patients with ovarian pathology such as endometrioma, dermoid or simple cyst or undergoing ovarian surgery were excluded from the study group. The diagnosis of HT was made with the presence of any of the anti-TPO, anti-TG thyroid autoantibodies diagnosed from the endocrinology and metabolic diseases clinic. All women with hypothyroidism were excluded from both groups.

Evaluation of the patients included in the study started with a history and physical examination. Patients’ age, pregnancy, birth and abortion counts, gynecological history, previous operations, presence of systemic disease (diabetes, hypertension, chronic liver and kidney disease, autoimmune diseases, etc.), family history, smoking, alcohol or drug use were questioned. Being fertile was defined a woman who has never had any problems with conceiving. In the first application of all cases, body mass index (BMI) was calculated in kg / m^2^ by measuring height (m) and weight (kg). Waist circumference was measured on the basis of navel level and hip circumference on the basis of large trochanter level. We classified phenotypes of PCOS patients according to the The Rotterdam consensus as follows: (A) hyperandrogenism (HA), oligo/anovulation and polycystic ovaries (PCO); (B) HA and oligo/anovulation; (C) HA and PCO; and (D) oligo/anovulation and PCO [17].

### Biochemical measurements

In the early follicular phase (days 2–5) of the menstrual cycle, 5 ml of blood samples were taken and Triglycerides (TG), Total Cholesterol (TC), Low Density Lipoprotein (LDL) and High Density Lipoprotein (HDL) fasting blood glucose, calcium, phosphorus levels were measured by enzymatic method (Beckman AU5800; Beckman Coulter Diagnostics, CA, USA). Follicle-stimulating hormone (FSH), luteinizing hormone (LH), estradiol (E2), progesterone, 17-hydroxyprogesterone (17-HOP), sex hormone binding globülin (SHGB), thyroid-stimulating hormone (TSH), free T3, free thyroxine (FT4), prolactin, hemoglobin, fasting insulin, vitamin D, anti-TG, anti-TPO, testosterone, DHEA-S levels were studied in Beckman Coulter DXI 800 device using the chemiluminescence method with Beckman Coulter commercial kits. The following formula was used to determine insulin resistance (HOMA-IR): fasting plasma insulin (mIU / L) x fasting plasma glucose (mmol / L) / 22.5. Values of 2.5 and above were considered as insulin resistance.

In order to study AMH, 2 ml venous blood samples were taken into biochemistry tubes that do not contain anticoagulant substance, 4000 rpm was centrifuged for 10 min and immediately collected in serum eppendorf tubes and stored at -80 °C until the day of analysis. AMH levels are measured using e commercially available ELISA kit (Sunred Biological Tecnology, Shanghai, China)(CV < 10%). The assays employed the quantitative sandwich enzyme immunoassay technique.

### Statistical analysis

Analyses were made with SPSS 18.0 package program. Descriptive statistics were presented with frequency, percentage, mean (mean), standard deviation (SD) and median (median), minimum (min), and maximum (max) values while evaluating the findings obtained in the study. Fisher’s Exact test or Pearson chi-square test was used to analyze the relationships between categorical variables. In the normality test, since the number of samples in the groups was less than 50, Shapiro Wilks test was used. Mann - Whitney U test was used in the analysis of the data that did not conform to the normal distribution between the measurement values of the two groups, and Student t test was used in the analysis of the data with normal distribution. Spearman correlation analysis was performed in cases where the normal distribution did not fit in determining the relationships between continuous variables. *P* values less than 0.05 were considered statistically significant.

## Results

In our study, 46 PCOS patients and 46 PCOS and HT patients were evaluated. The demographic characteristics of the PCOS and PCOS + HT cases are shown in Table 1.

**Table 1:** Demographic Data and Clinical Features of the Groups

Characteristics	PCOS (***n*** = 46)	PCOS + HT (***n*** = 46)		*P* value
Age, mean ± SD, years	26.4 ± 3.8	27.7 ± 3.2		0.072
Body mass index, mean ± SD, kg/m^2^	26.8 ± 5.4	27.1 ± 4.7		0.773
Waist/hip ratio, mean ± SD	0.75 ± 0.06	0.76 ± 0.07		0.737
Cigarette, n(%)				
No	22 (47.8)	25 (54.3)		0.532
Yes	24 (52.2)	21 (45.7)		
Chronic disease, n(%)				
No	39 (84.8)	29 (63)		0.060
Asthma	5 (10.9)	7 (15.2)		
Diabetes type 2	0	2 (4.3)		
Other	2 (4.3)	8 (17.4)		
Chronic Medication Use, n(%)				
No	38 (82.6)	29 (63)		0.035
Yes	8 (17.4)	17 (37)		
Menarche Age, mean ± SD, years	13 ± 1.1	13.2 ± 1.2		0.52
Menstrual Pattern, n(%)				
Regular	7 (15.3)	12 (26.1)		0.260
Oligo/ amenorrhea	37 (80.4)	33 (71.7)		
Polymenorrhea	2 (4.3)	1 (2.2)		
Acne, n(%)				
No	23 (50)	26 (56.5)		0.457
Yes	23 (50)	19 (41.3)		
Hirsutism Score (mean ± SD)	8.2 ± 4.8	8.5 ± 4.3		0.787
Fertility n(%)				
Fertile	20 (43.5)	8 (17.4)		0.007
Pr. infertile	15 (32.6)	29 (63)		
Sec. infertile	11 (23.9)	9 (19.6)		
İnfertility period, year±SD	2.2 ± 1.5	2.5 ± 2.7		0.477
Gravida	1 (0–4)	0 (0–3)		< 0.001
Parity	1 (0–3)	0 (0–3)		< 0.001
Abortion	0 (0–2)	0 (0–1)		0.14
Family history of autoimmune thyroiditis, n(%)				
No	35 (76.1)	20 (43.5)		0.001
Yes	11 (23.9)	26 (56.5)		
Family history of autoimmune disease, n(%)				
No	26 (56.5)	21 (45.7)		0.297
Yes	20 (43.5)	25 (54.3)		
HT period, year, median (min-max) mean ± SD	0	1.5 (0–10)	2.6 ± 2.9	
Thyroid Hormone Replacement (THR), n(%)				
No	46 (100)	20 (43.5)		< 0.001
Yes	0	26 (56.5)		
THR time, year	0	1 (0–16)	2.1 ± 3.2	

*SD* Standard deviation, *PCOS* Polycystic ovary syndrome, *HT* Hashimoto’s Thyroiditis

The ages of all patients were within the range of 18–35 years, the mean age was 26.4 ± 3.8 years in PCOS women and 27.7 ± 3.2 years in PCOS + HT women. While 26 (56.5%) patients in the PCOS + HT group had a family history of autoimmune thyroiditis, 11 (23.9%) patients in the PCOS group had a history of autoimmune thyroiditis in the family (*p* = 0.001). There was no difference between the two groups in terms of the incidence of other autoimmune diseases in the family (*p* = 0.297). While the number of patients with normal menstrual cycle in the PCOS + HT group was 12 (26.1%), it was 7 (15.3%) in the PCOS group. The most common form of menstrual irregularity in both groups was oligo / amenorrhea, and there was no difference in the rate of menstrual irregularity between the two groups (*p* = 0.26). While 20 (43.5%) patients in the PCOS group were fertile, 8 (17.4%) patients in the PCOS + HT group were fertile and there was a significant difference (*p* = 0.007). When evaluated in terms of hyperandrogenism, there was no significant difference in the presence of acne and hirsutism score according to the mFG scoring system (*p* > 0.05). There was no difference in age, BMI, waist / hip ratios of both groups (p > 0.05).

Laboratory data of the groups are summarized in Table 2. AMH values were significantly lower in the PCOS + HT group compared to the PCOS group (8.8 ± 8.8 vs 12.4 ± 8.1; *p* = 0.043). The mean AFC on both ovaries was significantly higher in the PCOS group (*p* = 0.034, *p* = 0.012). TSH values were 2.7 ± 2.1 in the group with PCOS + HT and 1.8 ± 0.9 in the PCOS group (*p* = 0.56); however, there was no significant difference in terms of free T3 and T4 hormones (*p* = 0.247, *p* = 0.369). No statistically significant difference was found between the two groups in terms of other laboratory parameters.

**Table 2:** Biochemical and hormonal parameters

Variable (mean ± SD)	PCOS (*n* = 46)	PCOS+HT (n = 46)	*P* value
TSH, μIU/mL	1.8 ± 0.9	2.7 ± 2.1	0.569
Free T3, pg/mL	3.2 ± 0.6	3.4 ± 0.7	0.247
Free T4, ng/dl	0.9 ± 0.3	1 ± 0.8	0.369
Anti-TPO, IU/mL	2.5 ± 2.7	248.3 ± 306.7	< 0.001
Anti-TG, IU/mL	1.03 ± 1.16	36.4 ± 62.3	< 0.001
Glucose, mg/dl	93.8 ± 20.1	95.1 ± 33.5	0.819
Fasting insulin, μIU/mL	24.3 ± 39.9	23.1 ± 31.7	0.877
Hemoglobin, g/dL	12.5 ± 1.04	12.2 ± 1.4	0.249
Hematocrit	38.03 ± 4.3	37.06 ± 3.8	0.259
FSH, mIU/mL	6.2 ± 1.7	6.8 ± 4.8	0.424
LH, mIU/mL	11.1 ± 7.1	7.5 ± 5.5	0.007
Estradiol, pg/mL	61.1 ± 33.9	57.2 ± 36.8	0.601
Progesteron, ng/mL	0.9 ± 0.8	0.7 ± 0.3	0.125
DHEA-S, μg/dl	342.5 ± 130.9	306.6 ± 141.5	0.211
17-OH Progesteron, ng/ml	2.05 ± 1.82	1.73 ± 1.01	0.308
Testosteron, ng/mL	0.8 ± 0.5	0.9 ± 0.5	0.44
Prolaktin, ng/mL	12.4 ± 6.6	11.02 ± 5.4	0.273
SHBG, nmol/L	26.9 ± 17.3	37.9 ± 34.7	0.058
Androstenedion, ng/mL	4.1 ± 2.5	4.3 ± 3.7	0.783
Vitamin D, ng/mL	14.7 ± 6.9	16.5 ± 12.6	0.413
HOMA-IR	5.3 ± 9.2	4.6 ± 6.6	0.67
Cholesterol, mg/dL	173.8 ± 33.5	177.2 ± 37.9	0.649
HDL-C, mg/dL	46.04 ± 9.7	47.4 ± 12.01	0.537
LDL-C, mg/dL	102.7 ± 28.8	106.4 ± 36.1	0.587
Triglyceride, mg/dL	131.01 ± 70.3	125.8 ± 83.7	0.749
Calsiyum, mg/dL	9.1 ± 0.4	9.2 ± 0.5	0.574
Phosphorus, mg/dL	3.1 ± 0.7	3.1 ± 0.8	0.759
Ovary Volume, mm^3^			
Right ovary	11.4 ± 3.8	10.08 ± 4.5	0.654
Left ovary	11.3 ± 4.9	10.3 ± 6.2	0.985
Antral Follicle Count (AFC), N			
Right ovary	14.2 ± 4.2	8.3 ± 4.6	0.034
Left ovary	14.8 ± 5.3	8.2 ± 4.1	0.012
AMH, ng/mL	12.4 ± 8.1	8.8 ± 8.8	0.043

*SD* Standard deviation, *PCOS* Polycystic ovary syndrome, *HT* Hashimoto’s Thyroiditis, *TSH* Thyroid-stimulating hormone, *Anti-TG* Anti-thyroglobulin, *Anti-TPO* Anti-thyroid peroxidase, *HOMA-IR* Homeostasis model assessment insulin resistance index, *HDL-C* Highdensity lipoprotein cholesterol, *LDL-C* Low-density lipoprotein cholesterol, *SHBG* sex hormone binding globulin, *DHEAs* dehydroepiandrosterones, *AMH* Anti-Müllerian hormone, *FSH* Follicle-stimulating hormone

**Fig. 1:**
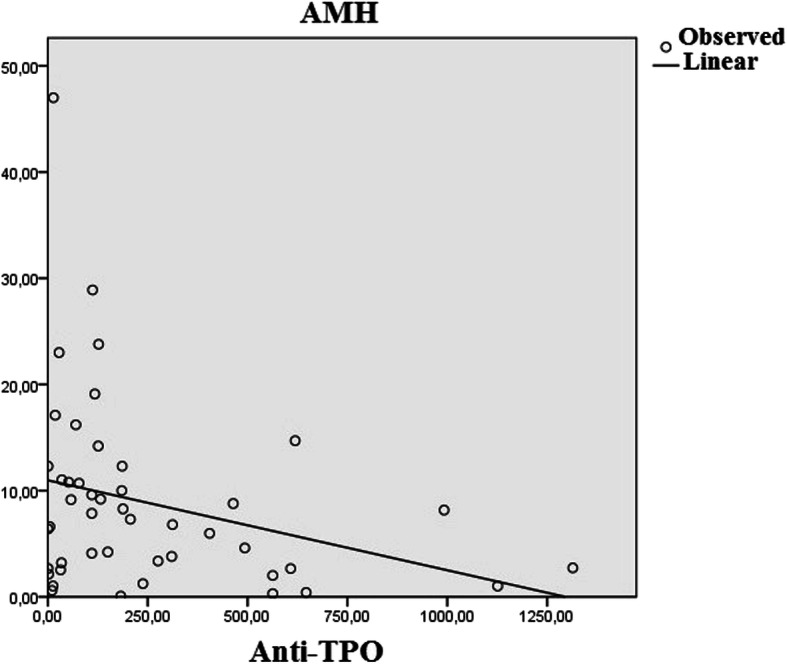
Correlation curve between AMH level and anti-TPO

**Fig. 2:**
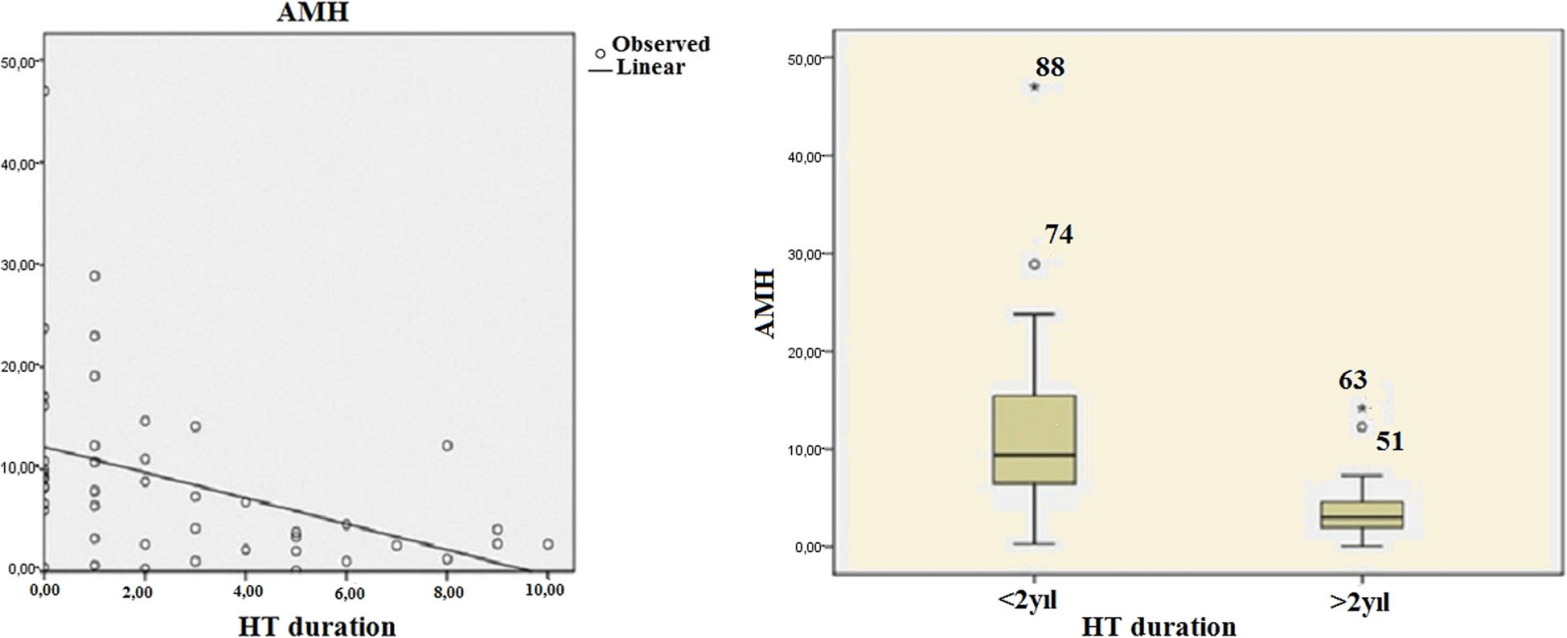
Correlation curve between AMH level and time after HT diagnosis

Correlation coefficients and significance values between AMH level and other parameters in both groups are given in Table 3. There was a significant negative correlation between anti-TPO and AMH serum levels in the PCOS + HT group (*r* = − 0.294, *p* = 0.047) (Fig. 1). No relationship was observed between anti-TG and TSH levels and AMH (*p* = 0.728, *p* = 0.246, respectively). A significant negative correlation was found between AMH level and the time elapsed after the diagnosis of HT (*r* = − 0.418, *p* = 0.004). Patients with HT (PCOS + HT group) were grouped as those with HT for less than 2 years and HT for more than 2 years and compared to the time elapsed after diagnosis (Fig. 2). While the mean AMH level was 12.2 ± 8.7 in patients with HT less than 2 years, the mean AMH level in patients with HT for more than 2 years was 4.2 ± 3.7 (*p* < 0.001). There was a significant positive correlation between the anti-TPO level and the time elapsed after the diagnosis of HT (*r* = 0.457, *p* < 0.001).

**Table 3:** Correlation coefficients and significance values between AMH level and other parameters

	PCOS (*n* = 46)		PCOS+HT (*n* = 46)	
	(r)	(p)	(r)	(p)
İnfertility time	0.220	0.143	0.248	0.097
HT duration			−0.418	**0.004**
Drug use period	−0.184	0.221	−0.284	0.056
Anti-TPO	0.038	0.804	−0.294	**0.047**
Anti-TG	0.146	0.332	−0.053	0.728

When both groups were compared in terms of PCOS phenotype frequency, the most common phenotype was found to be Type A, and no significant difference was found in both groups (*p* = 0.812).

## Discussion

PCOS is the most common hormonal disorder in women of reproductive age. Several studies suggest a role of autoimmunity in the pathogenesis of PCOS and women with PCOS have a 5-fold higher risk of HT [3, 18–20]. Another study reported a three-fold higher prevalence of HT in patients with PCOS [3, 8, 21]. Du and Li aimed to evaluate the relationship between PCOS and thyroid autoimmunity (TAI) by performing a meta-analysis of 6 studies involving 726 PCOS patients and 879 controls and they showed that HT and thyroid autoantibody levels were higher in PCOS [22]. Both PCOS and HT are assosiated with fertility problems in reproductive age women as infertility, miscarriage and may also cause complications as gestational hypertension, preeclampsia, preterm delivery, postpartum haemorrhage and lower birth weight [23, 24]. In our study, we proposed two hypotheses that have never been explored before; firstly, the ovarian reserve was evaluated with AMH levels and a lower ovarian reserve was observed in combination of both autoimmune diseases and secondly, it has been shown that as the duration of HT increases, ovarian reserve decreases due to the increase in autoantibody exposure.

Although the underlying pathophysiological mechanism of the association between thyroid autoimmunity and ovarian reserve status is not completely understood, many studies have reported high levels of autoantibodies against ovarian tissue, suggesting that autoimmunity may also be a component of pathophysiology [19, 25, 26]. For the first time Monteleone et al. reported the presence of thyroid antibodies in ovarian follicular fluid and in correlation with serum levels [27]. A possible mechanism may be anti-TPO passes through the blood follicle barrier during follicular evolution and that may result in destruction and damaging of growing follicles and oocytes [27]. So that thyroid antibodies seem to be having a direct impact on ovarian tissue. Bellver et al. showed higher prevelance of autoimmune thyroid disease in PCOS patients with unexplained infertility and implantation failure [28]. Recently, serum AMH has been accepted as a reliable marker for ovarian reserve. Several studies have analyzed the correlation of ovarian reserve in autoimmune thyroidism, assessed by serum AMH levels and TSH concentrations and/or thyroid autoantibodies and many of these studies demonstrated significantly higher levels of anti-TPO in infertile women with lower ovarian reserves with different reasons [12, 26, 29]. Chen et al. [30] reported that idiopathic low ovarian reserve with lower serum levels of AMH was associated with more frequent positive anti-TPO rather than thyroid function or anti-TG positivity, in a study of 1044 infertile Chinese women. In our study, patients with any antibody positivity were included in the PCOS + HT group, there were 18 patients (39.2%) with only anti-TPO positivity, 5 patients (10.8%) with only anti-TG positivity and 23 patients (50%) had both antibodies. AMH levels were similar in these three groups but significantly lower in PCOS + HT group. We pointed out that there was a significant negative correlation between anti-TPO and AMH serum levels in PCOS + HT group, AMH level decreased as anti-TPO level increased.

It is still unclear how thyroid autoimmunity can impair ovarian function and ovarian reserve but it is important to be specified to prevent ovarian insufficiency related thyroid autoimmunity. Sağlam et al. found lower AMH levels and lower pregnancy rates in euthyroid women with HT compared to the control group and women with HT were associated with premature ovarian insufficiency (POI) [31]. Among autoimmune disorders, autoimmune thyroid diseases are the most prevalent diseases associated with POI [10, 32]. Belvisi et al. reported at least one organ-specific autoantibody in women with POI with anti-thyroid autoantibodies most common (20%) [33]. Pogacnik et al. showed a strong correlation between HT and POI and reported a significant increase in anti-TPO and anti-TG levels compared to the control group [34]. It has been reported that thyroid autoimmunity is often diagnosed in patients with POI and the frequency of TPOAb was significantly higher (24.1%) in patients with POI [32, 34, 35]. In adolescents, when compared with the results of the studies conducted in women of reproductive age, the results were opposite. Erol et al. demonstrated that serum AMH levels were significantly higher in adolescents with Hashimoto’s thyroiditis than healthy adolescents [36]. Similarly, Pirgon et al. studied to determine the presence of antiovarian antibodies and the ovarian reserve effect of these antibodies in euthyroid adolescents with newly diagnosed HT, they reported higher AMH levels, also higher anti-TPO and anti-ovarian antibody levels in the group with HT than the control group [37]. In our study, the reproductive age group of 18–35 years old was evaluated and it was seen that AMH level was significantly lower in women with PCOS + HT compared to the PCOS group. So that it is thought that functional autoantibodies that cause hypo- and hyperfunction in the thyroid gland can be made the similar damage on ovaries and ovarian hyperfunction stage may be PCOS and the hypofunction stage may be POI [38]. Shamilova et al. compared the AMH levels of women with autoimmune POI and women with POI occured by another reason, and showed that women with autoimmune POI had a significantly higher AMH level [39]. It has been suggested that this is an early stage of autoimmune ovarian insufficiency [40]. These antibodies are thought to occur before clinical symptoms begin, and the possibility of predicting the future POI table in women with unexplained infertility is emphasized [10, 34, 37]. There are studies supporting this hypothesis, but the patient group who have both autoimmune diseases (PCOS+HT) has not been evaluated before. Also, the effect of the duration of autoimmune thyroiditis and duration of autoantibody exposure on the ovarian reserve is still unknown. In the current study, the presence of both antibodies in the PCOS + HT group and the lower AMH level suggests that the reduction in the ovarian reserve may be faster and more aggressive in these cases than the group with only PCOS.

Ott et al. underlined the relationship between PCOS patients with higher anti-TPO levels and inadequate treatment response in infertile patients treated with clomiphene citrate and metformin [26]. Magri et al. reported that women with autoimmune thyroid disease tend to respond poorly to controlled ovarian hyperstimulation with gonadotropins [41].

## Conclusıon

In this study, we pointed out the importance of exposure time of autoantibodies to high levels of autoimmune diseases together and we hypothesized that autoimmunity has a destructive effect on ovaries similar to thyroid gland as HT. Although thyroid hormone level assessment is essential for PCOS follow-up and treatment, thyroid autoantibody evaluation is mostly neglected. High autoantibody level is an important factor in insufficient response to infertility treatment. Because of all these close relations with PCOS and thyroid dysfunctions, we recommend evaluating both thyroid autoantibodies and hormone levels in PCOS patients at the first visit and euthyroid patients with positive autoantibodies should undergo screening closely for the possible thyroid disorders and related complications. Females with PCOS aged 28–35 years old in whom infertility is a difficult issue should be followed up on thyroid autoantibodies to offer better options. Future studies are needed to support the results of this research study and further investigate the role of underlying infertility diagnosis in the relationship of thyroid autoimmunity with female reproductive outcomes.

## Data Availability

The datasets used and/or analysed during the current study are available from the corresponding author on reasonable request.
